# Effects of iron deficiency on left ventricular functions in young women regardless of anemia: A speckle tracking echocardiography study

**DOI:** 10.55730/1300-0144.5370

**Published:** 2022-02-27

**Authors:** Betül CENGİZ ELÇİOĞLU, Onur BAYDAR, Alparslan KILIÇ, Nihal TEFİK, Füsun HELVACI, Erol GÜRSOY, Yasemin DEMİRCİ, Dilek URAL, Vedat AYTEKİN, Saide AYTEKİN

**Affiliations:** Department of Cardiology, Faculty of Medicine, Koç University Hospital, İstanbul, Türkiye

**Keywords:** Iron deficiency, subclinical left ventricular dysfunction, speckle tracking echocardiography, ferritin levels, heart failure

## Abstract

**Background/aim:**

Iron deficiency is one of the most common metabolic disorders worldwide and affects multiple organs and systems including the cardiovascular (CV) system. Iron deficiency can cause structural and functional changes in the myocardium. The aim of the study is to evaluate left ventricular (LV) functions in patients with low ferritin levels without anemia by two-dimensional “speckle tracking” echocardiography (2D STE).

**Materials and methods:**

We studied 90 participants (all female) that were divided into two groups according to ferritin levels (49 patients with ferritin levels <30 ng/mL, 41 age-matched controls with >30 ng/mL). Patients with anemia (hemoglobin level <12 g/dL), known CV disease, diabetes mellitus, low ejection fraction (<55%), active infection, high ferritin levels (>200 ng/mL) were excluded. All patients were evaluated by transthoracic echocardiography. In addition to conventional echocardiographic parameters and Doppler measurements, LV global longitudinal strain (GLS) and strain rate (GLSR) were obtained by 2D STE.

**Results:**

Mean ferritin level was 18.96 ± 7.29 ng/mL in low ferritin group, and was 61.22 ± 26.14 ng/mL in control group. There were no significant differences according to conventional and Doppler echocardiographic parameters between the groups. LV GLS and GLSR values were significantly lower in low ferritin group comparing with control group (17.31% ± 1.56 and 18.96% ± 1.53, p < 0.001; 0.64 ± 0.13 1/s and 0.81 ± 0.13 1/s, p < 0.001, respectively). There was a significant positive correlation between ferritin levels and LV GLS and GLSR values in study group (r = 0.482, p < 0.001; r = 0.387, p < 0.001, respectively). Ferritin level was also detected as an independent risk factor for GLS value < −18% in logistic regression analysis. In ROC curve analysis, the area under the curve for predicting GLS < −18% was 0.801 (p < 0.001, 95% CI 0.70–0.89) and the threshold of ferritin value was 28.5 ng/mL (sensitivity 76.1%, specificity 77.3%).

**Conclusion:**

Low ferritin levels can cause subclinical LV systolic dysfunction in patients without anemia. STE provides detailed information about LV functions. With larger studies, these patients should be followed more closely and considered for iron replacement treatment before developing anemia.

## 1. Introduction

Iron deficiency (ID) is the most common nutritional disorder and a major cause of anemia. While the prevalence of ID is 1%–4% in men and 5%–10% women in the general population, it is especially common among menstruating women with a prevalence of 9%–22% [1,2]. Iron is an important micronutrient involved not only in oxygen transport but also in many metabolic events [3]. A large amount of enzymes in the body requires iron for their functions. Iron also plays an essential role in cellular bioenergetics and mitochondrial metabolism.

Iron deficiency is a common comorbidity in patients with heart failure (HF) with a prevalence of up to 50% regardless of the presence of anemia and this coexistence is associated with poor prognosis [4,5]. Despite being such a common and important public health problem, iron deficiency is often overlooked and mostly left untreated unless anemia develops.

Low serum ferritin levels (<30 ng/mL) indicate ID regardless of anemia and are well known to be associated with adverse outcomes in patients with HF [6]. Since iron is an important component of mitochondria, its deficiency can cause impairment of mitochondrial metabolism in both skeletal myocytes and cardiomyocytes [7,8]. It has been shown in animal studies that ID causes structural changes in the myocardium and cardiac dysfunction [9–11]. It is thought that there may be a relationship between low ferritin levels and the development of HF independent from anemia and other cardiovascular (CV) risk factors.

Most of the studies investigating the relationship between ID and cardiac functions have been conducted in patients with anemia or in patients who already have had HF. The effect of low ferritin levels on heart functions in healthy subjects regardless of anemia has not been adequately studied. Speckle tracking echocardiography (STE) is a novel method that provides more detailed and reliable information about LV functions. The aim of this study is to investigate subclinical LV systolic dysfunction in childbearing age women with low ferritin levels without anemia.

## 2. Methods

### 2.1. Study population

We studied 90 patients (all female) who applied to the cardiology outpatient clinics with various symptoms, whose iron parameters and complete blood count (CBC) test were required, and who were scheduled for echocardiography between 2019 and 2021. The patients were divided into two groups according to their ferritin levels. While those with ferritin values < 30 ng/mL constituted the patient group, those with ≥30 ng/mL were determined as the control group. Patients with anemia (hemoglobin level < 12 g/dL), known cardiovascular disease (CVD), more than mild valvular heart disease, poor echocardiographic image quality, low ejection fraction (EF) (<55%), atrial fibrillation, and conduction abnormalities on electrocardiogram (ECG), diabetes mellitus (DM), active infection, malignancy, high ferritin levels (>200 ng/mL) and patients who have had iron replacement therapy or blood transfusion before were excluded.

Serum ferritin level was measured with electrochemiluminescence immunoassay (ECLIA) method using the Roche Cobas 6000 (Roche Diagnostics, Germany).

The study protocol was approved by the Local Ethics Committee of our institute, and a detailed written informed consent was obtained from each participant. The study was conducted according to the Declaration of Helsinki.

### 2.2. Echocardiographic assessment

Transthoracic echocardiography was performed in lateral decubitus position using Epiq 7C ultrasound system (Philips, Andover, MA, USA) equipped with a 2.3–3.5 MHz transducer probe with simultaneous ECG recording. Conventional measurements were made on images obtained from parasternal and apical windows after adjusting gain and frequency settings following the recommendations of the American Society of Echocardiography [12]. Left ventricular ejection fraction (LVEF) was calculated by the modified two-dimensional biplane Simpson’s method [13]. LV diastolic function was evaluated by trans-mitral velocities using pulsed wave Doppler and mitral annular velocities using tissue Doppler imaging (TDI).

**Figure 1:**
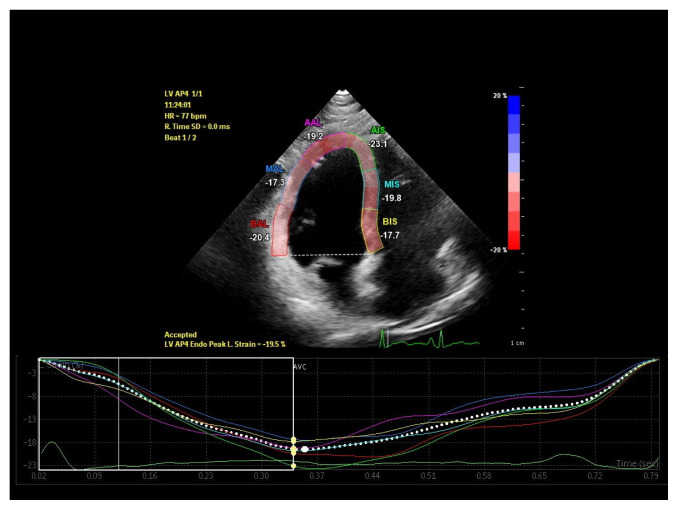
Left ventricular apical four-chamber global longitudinal strain imaging of a patient in low ferritin group.

**Figure 2:**
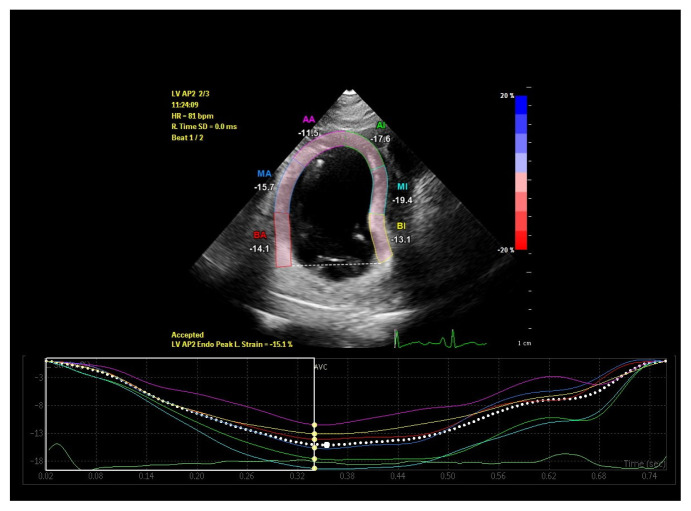
Left ventricular apical two-chamber global longitudinal strain imaging of a patient in low ferritin group.

**Figure 3:**
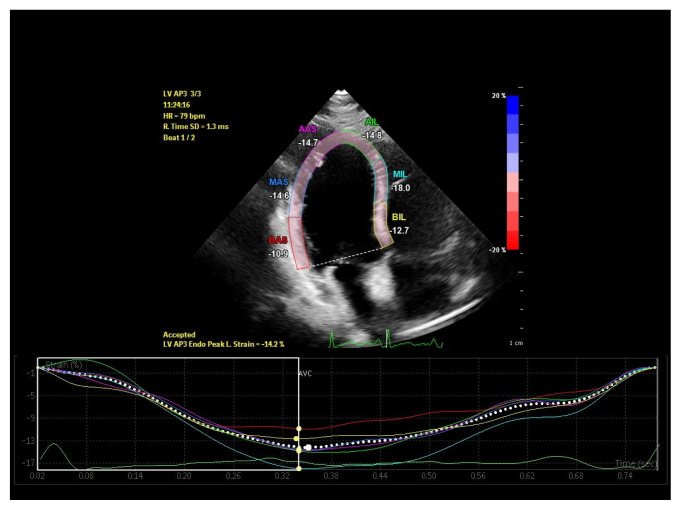
Left ventricular apical three-chamber global longitudinal strain imaging of a patient in low ferritin group.

**Figure 4:**
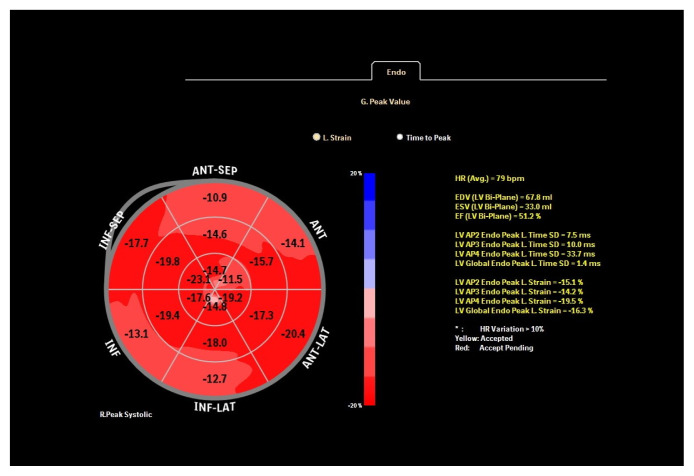
Left ventricular bull’s eye image of a patient in low ferritin group.

Standard apical two, three and four chamber images were recorded over 3 cycles in gray scale with a frame rate between 60–100 frames/s for two dimensional speckle tracking echocardiography (2DSTE) assessment. Offline LV strain analysis was performed using dedicated software (Qlab advanced quantification software version 10.1, Philips Medical Systems, Bothell, WA, USA). After tracing LV endocardial border manually at the end of the systole, the region of interest between endocardium and epicardium was created automatically by the computer. If necessary, the width and shape of ROI were adjusted manually to optimize tracking. Global longitudinal strain (GLS) and strain rate (GLSR) values were derived by taking the average of strain measurements obtained at three levels of six segments from each apical window (Figures 1–4).

## 3. Statistical analysis

Statistical Package for the Social Sciences 26.0 (SPSS, Chicago, IL, USA) program was used for the statistical data analysis. Kolmogorov Smirnov test was applied to test the normality of the distribution. The results were presented as means **±** standard deviations. Normally distributed continuous variables were compared using the Student t-test, and those not, were compared using Mann Whitney-U test. The chi-square test was used to compare categorical data. Statistical significance was defined as p-value less than 0.05. Correlation analyses were derived by using Pearson analysis for continuous variables and Spearman’s test for noncontinuous variables and correlation coefficient (r) was calculated. To evaluate the effects of various factors on GLS, we performed multivariate regression analyses using the Logistic Regression (LR) method. Receiver operating characteristic (ROC) curve analysis was used to determine the threshold of the ferritin value to predict LV GLS < −18%.

## 4. Results

Consecutive 90 female patients were enrolled in the study and classified into two groups according to ferritin levels (49 patients with ferritin levels < 30 ng/mL, 41 age-matched controls with >30 ng/mL). The demographic and clinical features of the study groups are presented in Table 1. The mean ferritin level was 18.96 ± 7.29 ng/mL in the low ferritin group and was 61.22 ± 26.14 ng/mL in control group. In addition, in the low ferritin group other biochemical and blood count parameters related to iron levels were found to be significantly lower (Table 2). There were no significant differences according to conventional and Doppler echocardiographic parameters between the groups. LV GLS and GLSR values were significantly lower in low ferritin group comparing with controls (−17.31% ± 1.56 and −18.96% ± 1.53, p = 0.0001; 0.64 ± 0.13 and 0.81 ± 0.131/s, respectively) (Table 3).

**Figure 5:**
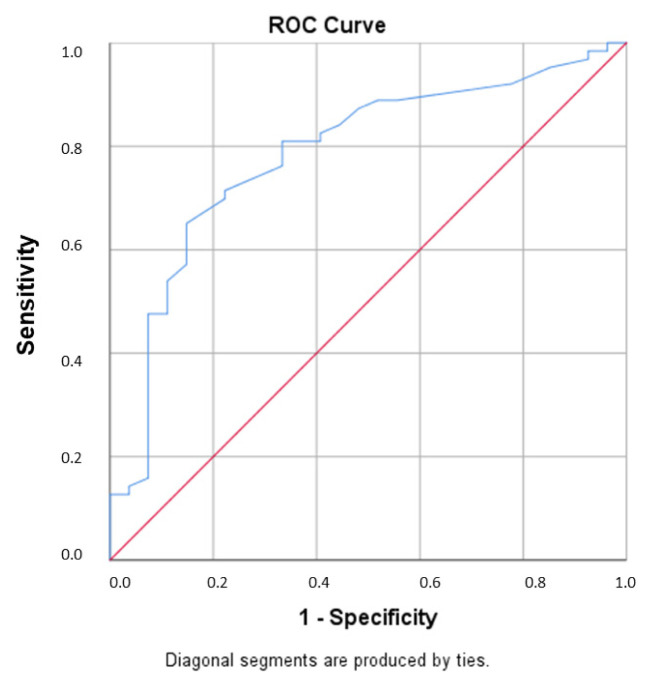
In receiver operating curve analysis, the area under the curve for predicting GLS < −18% was 0.801 (p < 0.001, 95% CI 0.70–0.89) and cut-off ferritin value was 28.5 ng/mL (sensitivity 76.1%, specificity 77.3%).

There was significant positive correlation between ferritin levels and LV GLS and GLSR values in study group (r = 0.482, p < 0.001; r = 0,387, p < 0.001, respectively). Ferritin level was also detected as an independent risk factor for GLS value < −18% in logistic regression analysis (Table 4). In ROC curve analysis, the area under the curve for predicting GLS < −18% was 0.801 (p < 0.001, 95% CI 0.70–0.89) and the threshold of ferritin value was 28.5 ng/mL (sensitivity 76.1%, specificity 77.3%) (Figure 5).

## 5. Discussion

The results we obtained in the study revealed that subclinical impairment of LV systolic function may develop in young women with low ferritin levels without anemia and overt CV disease.

The relationship between ID and HF has become the focus of researchers in recent years. Studies have mostly investigated the effects of IDA or the outcomes of low iron and ferritin levels in HF. Prevalence of iron deficiency in premenopausal women is approximately 10%–20% and only 2%–3% of those have anemia [14]. Iron deficiency can last for years before anemia develops and the diagnosis may be overlooked especially in asymptomatic individuals. In clinical practice, unless anemia develops, ID is paid insufficient attention by most clinicians and cannot become the main target of the treatment.

Serum ferritin level below 30 ng/mL is the most sensitive and specific test for the determination of iron deficiency [15]. The diagnostic value of ferritin decreases under conditions such as active infection, chronic inflammatory disease, and advanced age [16,17]. In a study by Silvestre et al. investigating the relationship between ferritin levels and the risk of developing HF, 1063 patients without HF have been followed for approximately 20 years. Both high and low ferritin levels have been associated with the development of new HF [18]. It has been shown in some animal studies that ID causes impairment in LV function independent of anemia [9,14]. Rineau et al. demonstrated in a study conducted on mice that ID without anemia was associated with reduced exercise capacity and LV function due to alteration of the mitochondrial function of cardiomyocytes. In addition, they demonstrated that both exercise capacity and LV dysfunction are reversed after iron treatment [10]. In a study by Hoes et al. examining the effects of iron deficiency on cardiac functions at the cellular level, it was shown that human embryonic stem cell-derived iron-depleted cardiomyocytes had reduced cellular ATP levels, impaired mitochondrial respiration, and contractile force. It was also observed that these effects reversed within three days with transferrin-bound iron supplementation [19].

The involvement of iron in myocardial metabolism as a part of oxidative enzymes and mitochondrial respiratory chain proteins plays a role in the impairment of cardiac functions even in the absence of anemia. Based on experimental studies and animal models, the early changes caused by iron deficiency in the heart muscle can be explained by the role of iron in the mitochondrial enzyme system and collagen synthesis. The decrease in collagen synthesis due to iron deficiency causes a change in the pressure-volume relationship with the reduced elasticity in the myocardium [20]. ID also affects mitochondrial bioenergetic functions resulting in mitochondrial swelling in myocardial cells, irregularities in sarcomere organization, disruption of the cell proliferation cycle, resulting in cessation of mitosis and apoptosis [21].

Strain analysis with speckle tracking echocardiography is a method that provides more detailed and accurate information about LV functions and has been used increasingly in studies and in clinical practice. Although impairment in left ventricular and atrial functions related to IDA has been demonstrated in some studies using STE [22,23], to the best of our knowledge, subclinical LV dysfunction has not been investigated in patients with low ferritin levels without anemia. With this study, we showed low ferritin levels are associated with low LV global longitudinal strain and strain rate values regardless of hemoglobin levels in young women. Also, we found a positive correlation between ferritin levels and LV GLS and GLSR measurements in the study group. The range of values considered normal for serum ferritin is quite wide. In this study the cut-off value of serum ferritin, predicting GLS value < −18% was found to be 28.5 ng/mL with ROC curve analysis. Additionally, in logistic regression analysis, low serum ferritin levels were shown to be an independent risk factor for LV subclinical dysfunction.

The major limitation of the study was that it included a small group of patients and was conducted only in women of childbearing age. This group was chosen because iron deficiency is a more common and important problem in young women. The results of the study need to be validated in different population groups. Since our study was only observational, whether the patients received iron replacement therapy afterwards and if they did, the results were not followed. Also, it was not known how long the patients were iron deficient.

In conclusion, iron deficiency is a metabolic disorder that affects large populations around the world. Before anemia develops, patients may remain undiagnosed for a long time and cellular morphological and functional abnormalities that develop during this period may cause subtle myocardial impairment before overt cardiac dysfunction occurs. Regular control of iron parameters becomes important, especially in young women and symptomatic individuals. With larger studies also investigating the efficacy of the treatment on cardiac functions, it may be considered to follow up iron-deficient patients more closely and with novel echocardiographic methods and to initiate iron replacement therapy at an early stage.

## Figures and Tables

**Table 1:** Demographic and clinical features of the study group.

Parameter	Low ferritin group (n = 49)	Control group (n = 41)	p value
**Age**	35.29 ± 9.05	38.76 ± 9.75	0.084
**SBP (mmHg)**	109.18 ± 12.22	111 ± 9.99	0.364
**DBP (mmHg)**	69.39 ± 7.68	71.75 ± 7.8	0.156
**Heart rate (beat/m)**	75.12 ± 10.15	72.09 ± 6.68	0.106
**BSA (m** **^2^** **)**	1.67 ± 0.13	1.70 ± 0.21	0.378
**Hypertension n (%)**	6 (12.2%)	8 (19.5%)	0.343
**Hyperlipidemia n (%)**	8 (16.3%)	8 (19.5%)	0.694
**Smoking n (%)**	7 (14.3%)	6 (15%)	0.924
**ACEI n (%)**	1 (8.2%)	4 (2.5%)	0.248
**ARB n (%)**	0 (0.0%)	3 (7.5%)	0.051
**Beta blocker n (%)**	1 (2%)	0 (0.0 %)	0.364
**Statin n (%)**	2 (4.1%)	0 (0.0%)	0.196

SPB, systolic blood pressure; DBP, diastolic blood pressure; BSA, body surface area; ACEI, angiotensin converting enzyme inhibitor; ARB, angiotensin receptor blocker.

**Table 2:** Biochemical iron and hematologic parameters of the study group.

Parameter	Low ferritin group (n = 49)	Control group (n = 41)	p value
**Ferritin(ng/mL)**	**18.96 ± 7.29**	**61.22 ± 26.14**	**<0.001**
**Iron (mcg/dL)**	**81.22 ± 34.01**	**99.44 ± 31.58**	**0.017**
**IBC(mcg/dL)**	**358.95 ± 40.17**	**306.47 ± 41.25**	**<0.001**
**Hemoglobin (g/dL)**	**12.73 ± 0.73**	**13.22 ± 0.7**	**0.002**
**Hematocrit (%)**	**38.78 ± 2.5**	**39.84 ± 2.08**	**0.034**
**TSAT (%)**	**22.71 ± 10.39**	**32.93 ± 11.47**	**<0.001**
**WBC count (K/uL)**	6.98 ± 1.52	7.27 ± 1.49	0.377
**NLR**	2.09 ± 0.64	1.86 ± 0.65	0.102
**Platelet count(K/uL)**	277.23 ± 56.32	257.2 ± 47.09	0.080

IBC, iron binding capacity; TSAT, transferrin saturation; WBC, white blood cell count; NLR, neutrophil lymphocyte ratio.

**Table 3:** Comparison of echocardiographic measurements of the groups.

Parameter	Low ferritin group (n = 49)	Control group (n = 41)	P value
**IVS (cm)**	0.82 ± 0.06	0.84 ± 0.05	0.068
**PW (cm)**	0.81 ± 0.05	0.83 ± 0.06	0.063
**LVEDD (cm)**	4.42 ± 0.2	4.49 ± 0.2	0.131
**LVESD (cm)**	2.83 ± 0.24	2.88 ± 0.2	0.374
**LV EF (%)**	61.35 ± 1.67	60.76 ± 1.57	0.091
**LA (cm)**	3.5 ± 0.17	3.57 ± 0.14	0.068
**RA (cm)**	3.4 ± 0.17	3.44 ± 0.17	0.302
**RV (cm)**	3.17 ± 0.2	3.18 ± 0.18	0.913
**E wave velocity (cm/s)**	89.8 ± 12	82.75 ± 15	0.457
**A wave velocity (cm/s)**	67.40 ± 16	0.73 ± 0.17	0.229
**E/A ratio**	1.3 ± 0.37	1.17 ± 0.26	0.173
**DT (msn)**	168.35 ± 22.11	175.5 ± 28.78	0.825
**IVRT (msn)**	84.75 ± 6.22	87 ± 6.88	0.131
**e′ wave velocity (cm/s)**	15.98 ± 2.87	14.81 ± 3.11	0.080
**E/e′ ratio**	6.17 ± 1.07	6.23 ± 0.82	0.406
**GLS (%)**	−**17.31 ± 1.56**	−**18.96 ± 1.53**	**<0.001**
**GLSR (1/s**)	**0.64 ± 0.13**	**0.81 ± 0.13**	**<0.001**

IVS, interventricular septal thickness; PW, posterior wall thickness; LVEDD, left ventricular end-diastolic diameter; LVESD, left ventricular end-systolic diameter; LV EF, left ventricular ejection fraction; LA, left atrium; RA, right atrium; RV, right ventricle; DT, deceleration time; IVRT, isovolumic relaxation time; GLS, global longitudinal strain; GLSR, global longitudinal strain rate.

**Table 4:** Independent risk factors of pathological GLS value in logistic regression analysis.

Variables	OR (95% C.I)	P
Age	1.0 (0.9–1.1)	0.32
Hypertension	2.2 (0.4–11.6)	0.33
Hyperlipidemia	1.9 (0.3–9.4)	0.42
Smoke	1.5(0.3–7.7)	0.57
Ferritin	0.93 (0.90–0.97)	0.001
